# “I didn’t mean it that way…”: Design and evaluation of an elective course on dealing with discrimination in medical communication

**DOI:** 10.3205/zma001764

**Published:** 2025-06-16

**Authors:** Lena Schwaab, Bernhard Strauß, Swetlana Philipp

**Affiliations:** 1 Friedrich Schiller University Jena, University Hospital Jena, Institute for Psychosocial Medicine, Psychotherapy and Psychooncology, Jena, Germany

**Keywords:** medical teaching, discrimination, simulation patients, communicative competence

## Abstract

**Objective:**

Experiences of discrimination in the context of medical care are not uncommon and have a significant impact on the health of those affected. For this reason, an elective course (28 units) on “dealing with discrimination in medical communication” was introduced at University Hospital Jena for medical students in the clinical section, which aims to improve the communicative skills of future doctors with marginalized patients. The course was tested for the first time in the winter semester 2023/24. The evaluation was used to check whether the course is suitable for expanding knowledge and skills in dealing with discrimination, as assessed by the students themselves.

**Methodology:**

The course includes discrimination-sensitive treatment of blind/visually impaired patients, trans*/non-binary patients, patients with right-wing extremist attitudes and/or conspiracy beliefs, people without health insurance, as well as racism- and trauma-sensitive treatment and the use of language mediation. The course also contained numerous elements for self-reflection and reflection on one’s own professional attitude as a doctor. The self-assessment of knowledge and competence gains is based on an online survey at the beginning of the first course and at the end of the last session.

**Results:**

The participating students (N=13) had hardly had any contact with the content of the seminar series through their medical studies, although they rated the relevance for their own practical work as high. The evaluation of the pre- and post-survey showed that completing the course led to significant increases in self-assessed knowledge for all course topics. There was an increase in self-assessed competence, particularly in relation to dealing with blind/visually impaired people, traumatized people and the use of interpreters. The ability to self-reflect and deal with one’s own weaknesses, as well as the awareness of recognizing discrimination, was rated significantly higher also.

**Conclusion:**

The newly designed elective subject could help to subjectively improve medical students’ knowledge and skills in dealing with discrimination in medical communication. The findings thus provide a good basis for the development of further teaching concepts.

## Zusammenfassung

**Zielsetzung:**

Diskriminierungserfahrungen im Rahmen der medizinischen Versorgung sind keine Seltenheit und beeinflussen maßgeblich die Gesundheit Betroffener. Daher wurde am Universitätsklinikum Jena ein Wahlfach (mit 28 UE) für Medizinstudierende im klinischen Abschnitt zum Thema „Umgang mit Diskriminierung in der medizinischen Kommunikation“ eingeführt, welches die kommunikativen Kompetenzen der angehenden Ärzt:innen mit marginalisierten Patient:innen verbessern soll. Der Kurs wurde erstmals im Wintersemester 2023/24 erprobt. Mit der Datenerhebung wurde überprüft, ob der Kurs geeignet ist, Wissen und Kompetenzen, welche von den Studierenden selbst eingeschätzt wurden, im Umgang mit Diskriminierung zu erweitern.

**Methodik:**

Die Lehrveranstaltung beinhaltet den diskriminierungssensiblen Umgang mit blinden/sehbehinderten Patient:innen, trans*/nicht-binären Patient:innen, Patient:innen mit rechtsextremen Einstellungen und/oder Verschwörungsglauben, Personen ohne Krankenversicherung, sowie rassismus- und traumasensible Behandlung und den Einsatz von Sprachmittlung. Zudem enthielt der Kurs zahlreiche Elemente zur Selbstreflexion und Reflexion der eigenen professionellen Haltung als Ärzt:in. Die Selbsteinschätzung des Wissens- und Kompetenzzuwachses basiert auf einer Online-Erhebung zu Beginn der 1. Veranstaltung und zum Abschluss der letzten Sitzung.

**Ergebnisse:**

Die teilnehmenden Studierenden (N=13) hatten bisher durch ihr Medizinstudium kaum Kontakt zu den Themeninhalten der Seminarreihe, obwohl sie die Relevanz für die eigene praktische Arbeit als hoch einschätzten. Die Auswertung der Prä- und Posterhebung ergab, dass die Absolvierung des Kurses zu deutlichen Zuwächsen im selbst eingeschätzten Wissen bei allen Kursthemen führte. Eine Steigerung der selbst eingeschätzten Kompetenz erfolgte vor allem in Bezug auf den Umgang mit blinden/-sehbehinderten Personen, mit traumatisierten Personen und den Einsatz von Dolmetschern. Auch die Fähigkeit zur Selbstreflexion und den Umgang mit eigenen Schwächen, sowie die Sensibilisierung für das Erkennen von Diskriminierung wurde signifikant höher eingeschätzt.

**Schlussfolgerung:**

Das neu konzipierte Wahlfach könnte helfen, das Wissen und die Kompetenzen im Umgang mit Diskriminierung in der medizinischen Kommunikation bei Medizinstudierenden subjektiv zu verbessern. Damit liefern die Erkenntnisse eine gute Grundlage für die Entwicklung weiterer Lehrkonzepte.

## 1. Background

Discrimination is a frequently discussed topic in medicine and healthcare. Based on a pluralistic definition, it is understood as the addition of harm, a violation of freedom rights, a disregard, and/or an inequality of opportunity towards people due to their (supposed) membership of certain social groups [1]. With the Geneva declaration, doctors commit themselves to ensuring that such social categories do not interfere with their duties and their patients [2]. Nevertheless, findings indicate that people in the healthcare sector experience discrimination due to disabilities, impairments and/or chronic illnesses, their gender identity (especially trans* and inter-persons), their ethnic origin or due to racial discrimination, their sexual orientation, their age, their religion, their weight or insufficient language skills [3], [4], [5], [6], [7], [8], [9]. Experiences of discrimination can significantly damage the relationship of trust necessary for successful treatment [3], [4]. An example of this is the representative National Discrimination and Racism Monitor 2023, which shows that racially marked women in particular (Black: 14.3%, Muslim: 12.8%, Asian: 12.9%) delay or avoid medical treatment due to the fear of not being taken seriously or being treated worse than others [10]. This also highlights the intersectional nature of discrimination, where multiple dimensions can lead to disadvantages based on very individual experiences [1], [3]. It should be emphasized that discrimination and sexual harassment also occur in medical teaching [11], [12], but this is not the focus of the present study.

In addition to barriers and inhibitions in accessing healthcare, the consequences of such unequal treatment in the medical field include inadequate treatment, misdiagnosis, lower compliance and satisfaction, and psychological impairment [6], [7], [13]. Discriminatory behavior, such derogatory remarks, verbal abuse, and addressing accompanying persons instead of patients, occurs particularly at the level of communication and interaction [3]. 

To counteract this, it is necessary to provide appropriate specialist knowledge along with sensitization and self-reflection concerning one's own position, power, privileges, inequality and different worldviews. However, this is often insufficient [3], [5], [14], [15], [16]. Surveys on the implementation of these topics in medical education indicate that the content predominantly focuses on “culture”, with such topics as gender, sexual and gender orientation (lesbian, gay, bisexual, trans, queer, inter, LGBTQI+), age and disability being inadequately addressed [17], [18], [19], [20], [21]. In Germany, medical education particularly lacks LGBTQI+-specific content [7], and intersectionality is often neglected [22], [23]. The teaching concepts presented in the literature to date consist of individual lectures [24], [25] or elective courses, typically completed in blocks of several hours [26], [27] or over an entire semester [27]. These courses address the handling and treatment of specific groups of people, such as trans* people [24], [28], refugees [25] and deaf patients [26], as well as language mediation [27]. Language mediation refers to interpreting activities that can be carried out by professional interpreters or lay interpreters such as untrained healthcare staff or relatives. The intersection of different forms of discrimination is occasionally discussed [17], [29]. Effective elements for implementing discrimination-sensitive content in medical education include the inclusion of multidisciplinary perspectives through cooperation with practitioners or the community (community-based teaching) [24], [25], [27]. Additionally, demonstrating that it is not about “the others” (othering), but about a differentiated examination of power, inequality, and privilege and their effects on one’s actions [17]. Closely linked to this is the development of a professional medical identity, where medical students reflect on and train the various aspects of medical practice [30]. Finally, the use of simulated patients has proven effective, allowing students to engage with challenging communicative situations in a protected environment [28], [31], [32], [33].

Based on these three elements, the elective course “I didn’t mean it that way ... - dealing with discrimination in medical communication” was conceptualized at University Hospital Jena, piloted in the winter semester 2023/24, and subsequently evaluated. The aim of this paper is to present this approach in order to make the entire concept or individual modules usable for other locations.

## 2. Teaching concept

### 2.1. Course concept and learning objectives

The elective course aims to address the different realities of life and experiences of discrimination of different groups of people, with a particular focus on communicative aspects. An extensive literature review of existing research in relation to discrimination-sensitive teaching and prior teaching concepts was integrated into the content of the elective course. In addition, an assessment was obtained from employees regarding relevant topics and practical requirements, as well as the wishes and needs of students regarding topics that had not yet been sufficiently covered in medical studies or had been addressed only in extracurricular contexts. Building on this, cooperation was established with various (local) institutions and advice centers. The individual subject areas of the seminars were designed by these experts (see table 1 (Tab. 1)). 

The specific learning objectives of the course (see attachment 1 ) can be found in the National Competence-Based Learning Objectives Catalog 2.0 [https://nklm.de/zend/menu].

Throughout the sessions, the person responsible for the course repeatedly made connections between the individual contents and discussed the phenomenon of intersectionality. The course comprised six sessions with a total of 28 teaching units. Twenty students from the 7^th^ and 9^th^ semesters were able to take part in the elective. An example of a session can be found in attachment 2 .

### 2.2. Main features of the course

Communication and social skills are trained primarily through *practical exercises*. For this reason, a variety of case studies and role plays were used in the sessions. For example, in the session on different approaches to healthcare, a simulation game was carried out in which students took on the role of patients with different socio-demographic backgrounds and conditions (e.g. no health insurance, unclear residence status) and experienced firsthand how healthcare system touchpoints responded to them. Dealing with a patient with right-wing extremist attitudes, who discusses these in an emotional way during treatment, and the use of language mediators (interpreters) was practised with simulated patients.

In order to develop measures that counteract discriminatory behavior, it is essential to consider the intention of the action [1]. The elective focused on *reflection and self-reflection* on non-intentional discriminatory actions. Self-reflection and professional identity development are particularly important for ethical issues that are closely linked to discrimination and medical responsibility. For example, trans* patients can question basic assumptions about gender, therefore it is necessary for practitioners to reflect on their own gender identity and role perceptions [6]. For this reason, elements of self-reflection were introduced in the very first session and discussed at the end of each session in individual work, in small groups and in plenary (see attachment 2 ).

### 2.3. Evaluation

To evaluate the course, a survey of the participating students was conducted both during the first (pre-survey) and the last session (post-survey) using the online tool SoSciSurvey. The pre- and post-data were assigned using a code chosen by the participants themselves. In addition to socio-demographic questions on age, gender and current semester, participants were then asked about their previous experience in the content topics on a five-point Likert scale. These questions referred to previous experience from medical studies as well as professional and non-specialist contexts (1=no experience, 5=very much experience). As part of the pre-survey, students were also asked about their expectations and specific questions that they wanted answered during the seminar.

The evaluation followed the approach of Juscyzk et al. [34]; in both the pre- and post-survey, students were asked to assess their attitudes, previous level of knowledge, interest, motivation, relevance and competence concerning the subject matter (see attachment 3 and attachment 4 ). The competency questions were based on the NKLM 2.0. Participation in the surveys was voluntary and anonymous; non-participation was not traceable to individual persons. Data were analyzed descriptively and inferentially (t-test for paired samples) using SPSS software (IBM SPSS 29), with the significance level set at α=0.05. Changes from pre to post were also presented using standardized mean differences and the spread of the difference values (effect size Cohen’s d, standardized response mean) [35], [36], with positive values indicating a gain. Additionally, a feedback round was conducted in the final session, where students – initially anonymously – noted particularly positive aspects of the elective and aspects that could be improved, which were then discussed in a plenary session.

## 3. Results

### 3.1. Socio-demographic data

Of the 13 participating human medicine students, two thirds (n=8) were in their 7^th^ semester, one was in the 9^th^ semester and two were in their 10^th^ or higher semester. The average age was 24.25 years (*SD*=2.67). The majority of students were female (83.3%), with 16.7% male. Twelve students each took part in both the pre- and post-surveys, with n=11 having available responses for both.

### 3.2. Previous experience with the seminar content

The pre-survey regarding the students’ previous experience with the topics covered in the seminar series revealed that they had the fewest points of contact with patients with communication impairments (no experience: 63.8%) and patients without health insurance (no experience: 61.1%). The majority had already had experience with refugees (69.4%) and with right-wing extremist patients and believers in conspiracies (69.1%). Looking at the contexts in which the previous experiences were made, it is striking that the students came into contact with the topics the least through their medical studies (37.5%). The students gained more experience through professional experience (e.g. internships, work shadowing, ward days) (47.2%) and in non-specialist contexts such as everyday life, leisure time, voluntary work (63.9%). 

### 3.3. Pre-post comparison

As part of the pre-post comparison, the development of the students’ interest, attitudes, knowledge and skills over time was examined.

The students’ *interest *in the individual topics of the course series was already high at the beginning of the course and for the most part did not change significantly towards the end of the course (see table 2 (Tab. 2)).

Significant changes in the *attitude* dimension were found for professional interaction with right-wing extremists (d=1.09), interaction with visually impaired/blind patients (d=0.92), the inclusion of relatives or friends as interpreters (d=0.80), and communication with people whose gender is not clearly categorized (d=0.80). The *attitudes* regarding the importance of dealing with different realities of life, knowledge of one’s own needs and boundaries, the perception of racist violence and discrimination as traumatization received similarly high approval ratings as at the beginning of the seminar (see table 3 (Tab. 3)).

Before the course the majority of students stated that they were hardly or only partially informed about the various contents of the seminar series. After the course, however, they stated that they were well informed in almost all subject areas (see table 4 (Tab. 4)). There were large effects for the change in *subjectively assessed knowledge about communicative strategies in dealing with blind/visually impaired people* (d=2.46) and knowledge* about central symptoms after traumatic ev*ents, their effects and the needs of those affected (d=2.22).

**Figure 1:**
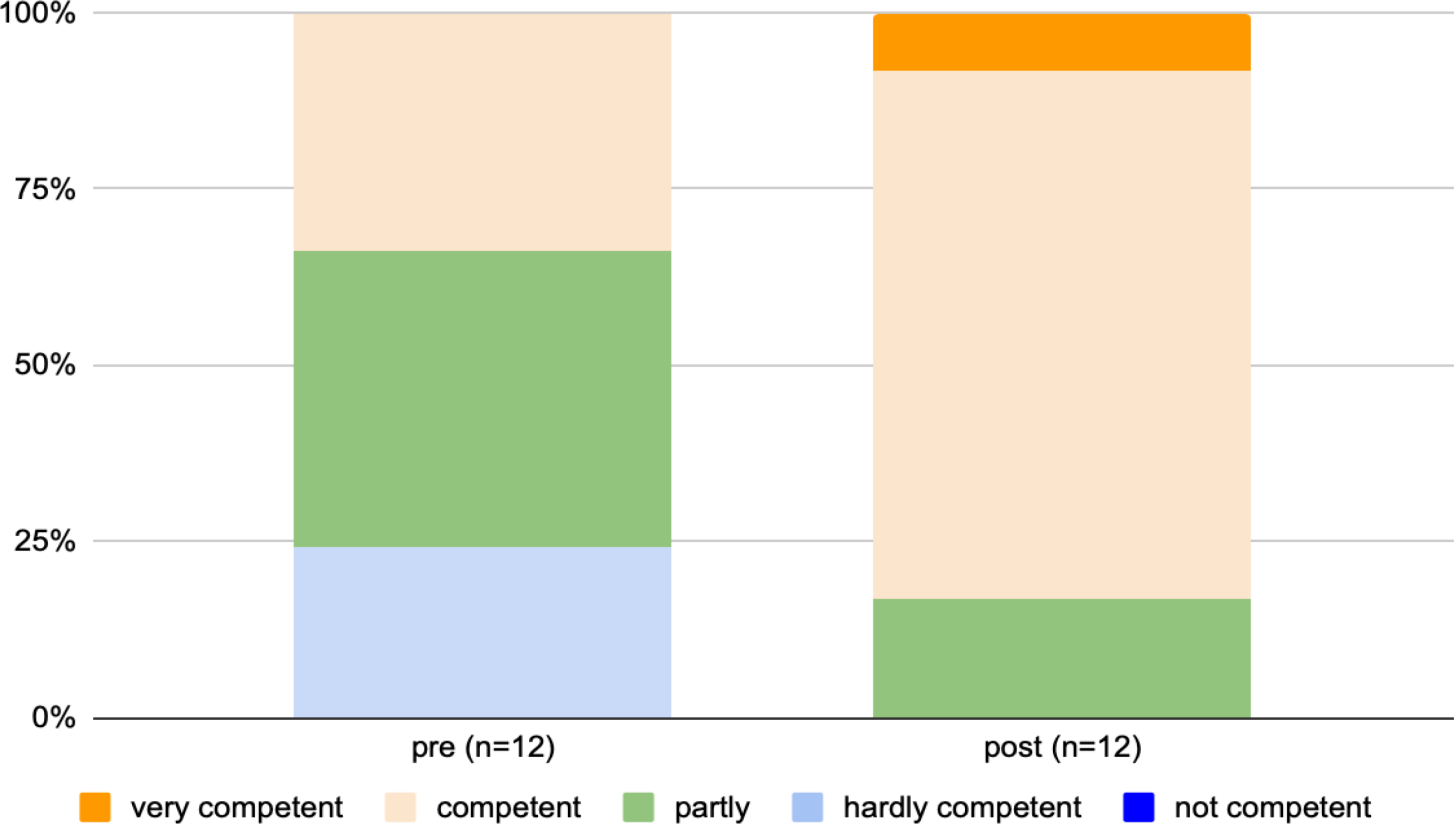
Subjective assessment of competence in recognising disadvantage, stigmatisation and discrimination, and orientation of one's own actions in terms of preventing or eliminating these disadvantages

The results presented in table 5 (Tab. 5) show that the students consider themselves to be significantly more competent in some areas after the seminar. The recognition of discrimination and the orientation of one’s own actions in terms of prevention and disadvantage should be emphasized here, as the learning of this competence spanned the entire seminar series (see figure 1 (Fig. 1)). The students rated themselves as significantly more competent in this area (d=0.92).

The effects of increased competence with regard to the consideration and use of relevant influencing factors in the interpreted conversation (d=1.30), the use of communicative strategies in dealing with blind/visually impaired patients (d=1.45), and the recognition of and reaction to symptoms of trauma-related disorders (d=1.65) can also be classified as high.

### 3.4. Evaluation of the course 

Overall, the course was rated by the students with an average grade of 1.75 (*SD*=0.42) on a 5-point Likert scale; 76.9% fully agreed with the statement that participation was worthwhile for them. The relevance of the content topics for practical work was rated as high on the four-point scale (1=not relevant, 4=very relevant) (*M*=3.22-3.83, *SD*=0.39-89). In the qualitative survey and in the open exchange, the students rated the selection of content, the various experts and their own connection to the community, as well as the use of practical exercises with the simulation patients, positively. The simulation game was particularly praised. The participants felt that the timing of the seminar could be improved, as the sessions took place late on Friday afternoons. They would also like to see more practical exercises.

## 4. Discussion

This article describes the design and evaluation of an elective course on dealing with discrimination in medical communication. To our knowledge, such a course, in which various forms of marginalization and discrimination are dealt with, has not yet been conceptualized and implemented. In addition, the medical students in Jena reported that these topics have hardly been addressed in their studies to date, even though they are highly relevant to their practical work. The results of the pre-post comparison of the survey data showed that the seminar series led above all to subjective increases in knowledge in relation to all the topics covered and to subjective increases in competence in the use of language mediation, in dealing with blind/visually impaired patients, and with traumatized persons. Higher-level skills, which included self-reflective skills and sensitization to discrimination, were also significantly improved according to the participants?’ self-reports. One possible explanation for the fact that primarily the participants’ assessed knowledge improved, with slightly fewer corresponding skills, may be the formulation of the skills items. These are based on the learning objectives from the NKLM 2.0 and are therefore partly formulated in a somewhat more general way and therefore do not exactly match the content covered in the seminar series. For example, there is no competence in the NKLM 2.0 that explicitly refers to dealing with trans* and non-binary people. For this reason, the learning objective (VIII. 2.05.1.2), which addresses gender-sensitive communication, was used. In addition, the acquisition of practical, communicative and social skills is a longer and difficult to measure process, which should also be considered beyond the seminar series. The complexity of competence acquisition and its measurement can only be referred to briefly at this point [37]. 

Due to selection effects, it is likely that those students who were already interested in and engaged with the subject content registered for the seminar. This could explain why the values for interests and attitudes were already high before the seminar. It is interesting to note that the significant changes in attitudes were seen in the items that were negatively polarized (e.g., “I find it difficult to communicate with patients whose gender I cannot clearly classify”), so that these changes can be interpreted as a reduction in inhibitions.

One limitation is that the evaluation of the course is based solely on the students’ self-assessment, and no objective test was used to change the level of knowledge or skills. The post-survey was also only conducted in the last session, meaning that long-term effects of the course cannot be considered. In addition to the subjective assessment of the students, it could be useful for future teaching evaluations to operationalize the changes in social skills by means of external assessments and to use further questionnaires that, for example, record the professional identity formation and thus the individual maturation process towards one’s own medical identity [30], [38]. It is also regrettable that the seminar was not fully utilized, and the number of participants on the individual dates fluctuated between 11-15 people, meaning that the data comparison is based on a relatively small sample. The students cited the seminar's unattractive scheduling structure as a possible reason, which must be taken into account when continuing the course. The gender ratio of the course, with significantly more female participants, does not quite reflect the ratio in medical studies in Jena, meaning that more female students generally seem to be interested in the subject content. This is also consistent with other courses that address diversity and discrimination [14], [26], [34].

Discrimination in healthcare is such a vast topic that a seminar series of this kind cannot cover all relevant issues exhaustively. Other possible topics, based on empirical findings, could include age, disability, weight, religion, socio-economic status, weight, and sexism in healthcare [4], [17]. However, the strength of such a seminar series, which addresses various forms of discrimination, lies in its overall sensitization to discrimination and in the development of a professional attitude that facilitates future contact with groups of people with whom doctors have had little prior interaction. Based on Paul Mecheril’s paradoxically formulated “competence-less competence” [18], a reflexive relationship to the conditions and consequences of their own professional actions as well as an open approach to their own ignorance and uncertainties could be initiated among the medical students. At this point, the following statement by one interviewee should be highlighted: *“Being open, self-reflective, curious and respectful – that is important when dealing with all marginalized group”*.

## 5. Conclusion

The results indicate that the communicative handling of discrimination in medical teaching has so far been insufficiently implemented, although there appears to be a high demand for it among students. The conceptualized elective provides initial starting points for filling this gap and led to self-reported changes among medical students, such as an increase in knowledge, a reduction in inhibitions, and the acquisition of higher-level skills such as a self-critical attitude and sensitization to discrimination. This work therefore offers starting points for new teaching concepts at other locations. The longitudinal implementation of the content in the compulsory curriculum would be desirable to promote a self-reflective attitude and the acquisition of professional, social, and communicative skills related to intersectional discrimination and its effects among as many students as possible.

## Notes

### Funding

The financing of the lecturers' fees was supported by the Förderverein Fachschaft Medizin e.V..

### Profile

- **Name of the location:** Jena
- **Subject/professional group:** Human medicine
- **Number of apprentices per year:** 286
- **Has a longitudinal communication curriculum been implemented? **partially
- **In which semesters are communication and social skills taught? **Compulsory in the 2^nd^, 3^rd^, 4^th^, 7^th^, 8^th^ and 10^th^ semesters as well as optional elective courses in preclinical and clinical courses
- **Which teaching formats are used?** Small groups with and without simulated patients, skills lab courses
- **In which semesters are communicative and social skills tested (formative or pass-relevant and/or graded)? **4^th^, 8^th^ and 10^th^ semester
- **Which examination formats are used?** OSCE
- **Who (i.e. clinic, institution) is entrusted with the development and implementation?** Contact person for Longitudinal Communication Curriculum: Dr. Swetlana Philipp, Contact person in the Dean of Studies Office: Christian Seidler, Contact person in the Skillslab: Urte Mille

## Competing interests

The authors declare that they have no competing interests. 

## Supplementary Material

Learning objectives of the course based on the National Competence-Based Learning Objectives Catalog 2.0

Example of a session

Pre-evalatuation questionnaire

Post-evaluation questionnaire

## Figures and Tables

**Table 1:**
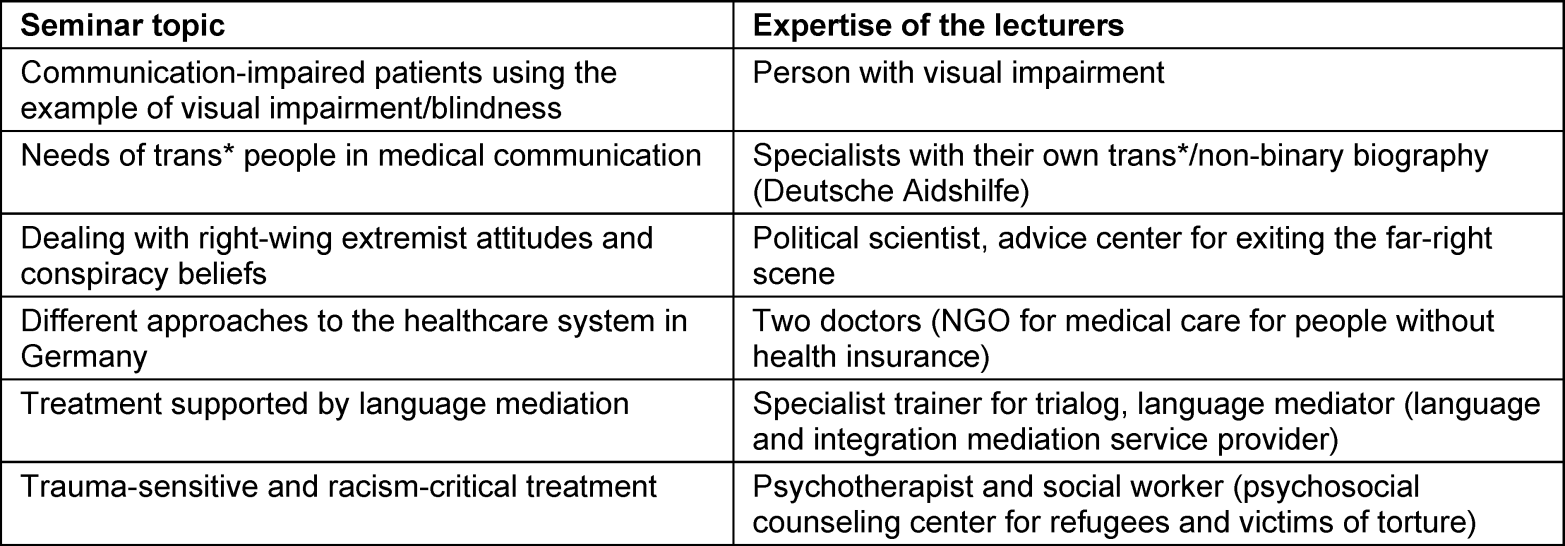
Seminar topics and experts

**Table 2:**
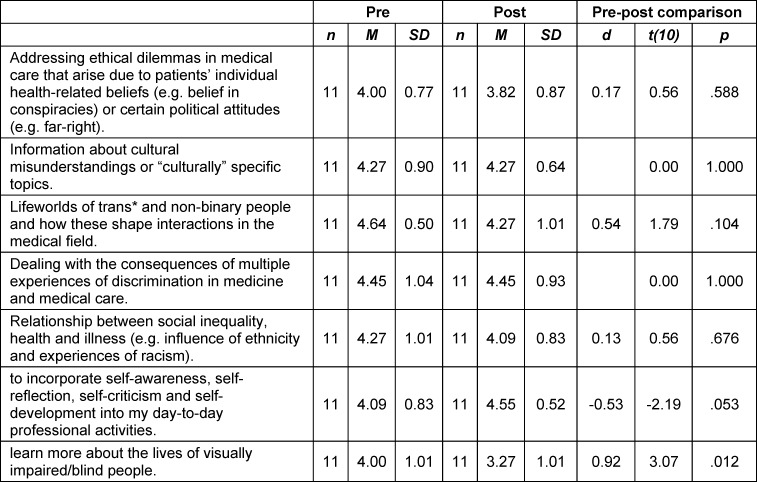
Subjective assessment of students’ interest before and after participating in the course, measured on a 5-point Likert scale (1=not interested, 5=very interested)

**Table 3:**
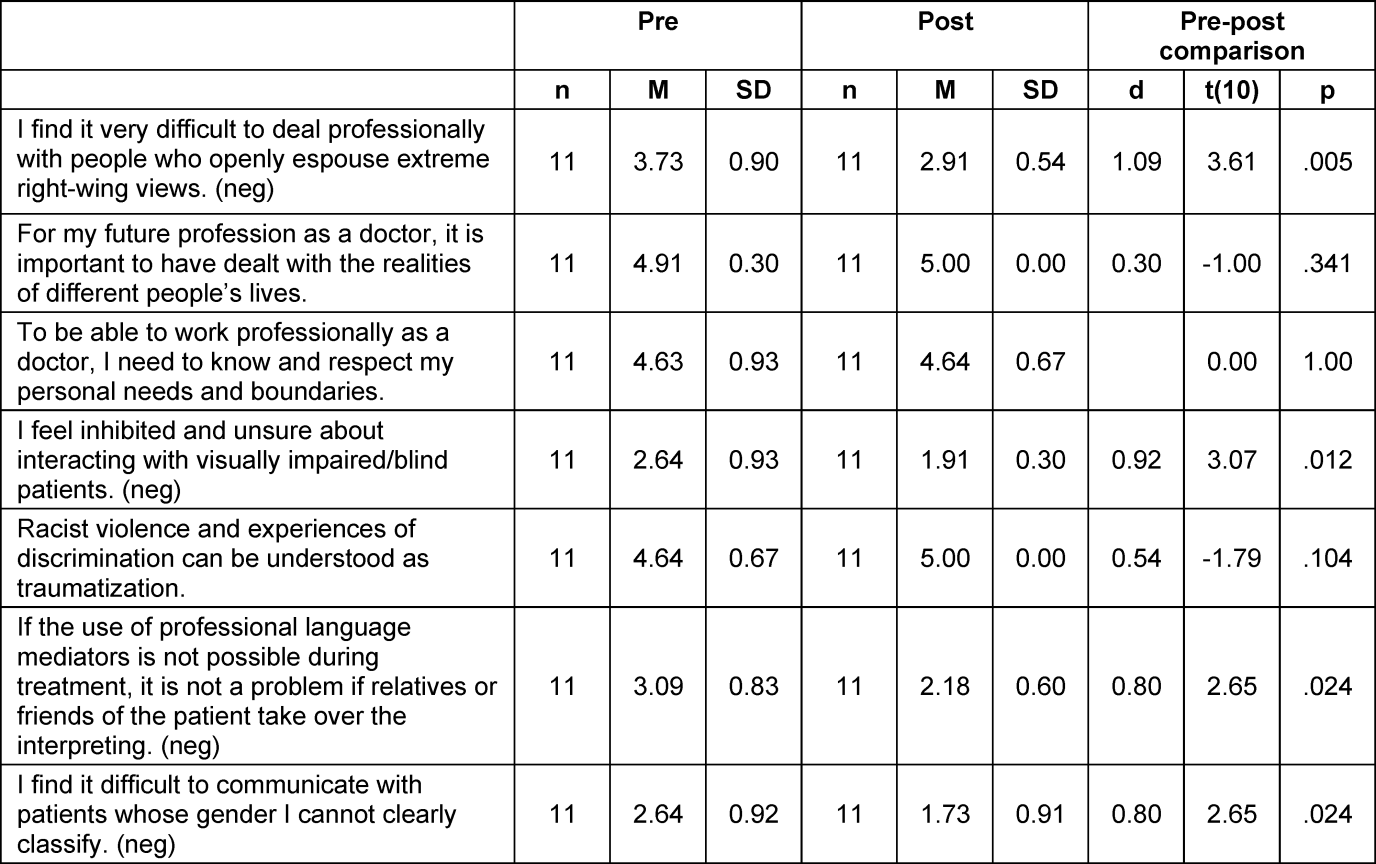
Students’ attitudes before and after participating in the course measured on a 5-point Likert scale (1=strongly disagree, 5=strongly agree)

**Table 4:**
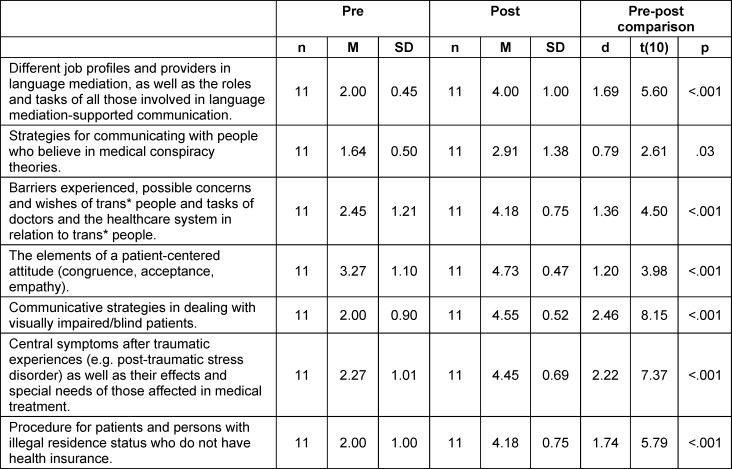
Subjective assessment of students’ knowledge before and after participating in the course, measured on a 5-point Likert scale (1=not informed, 5=very informed)

**Table 5:**
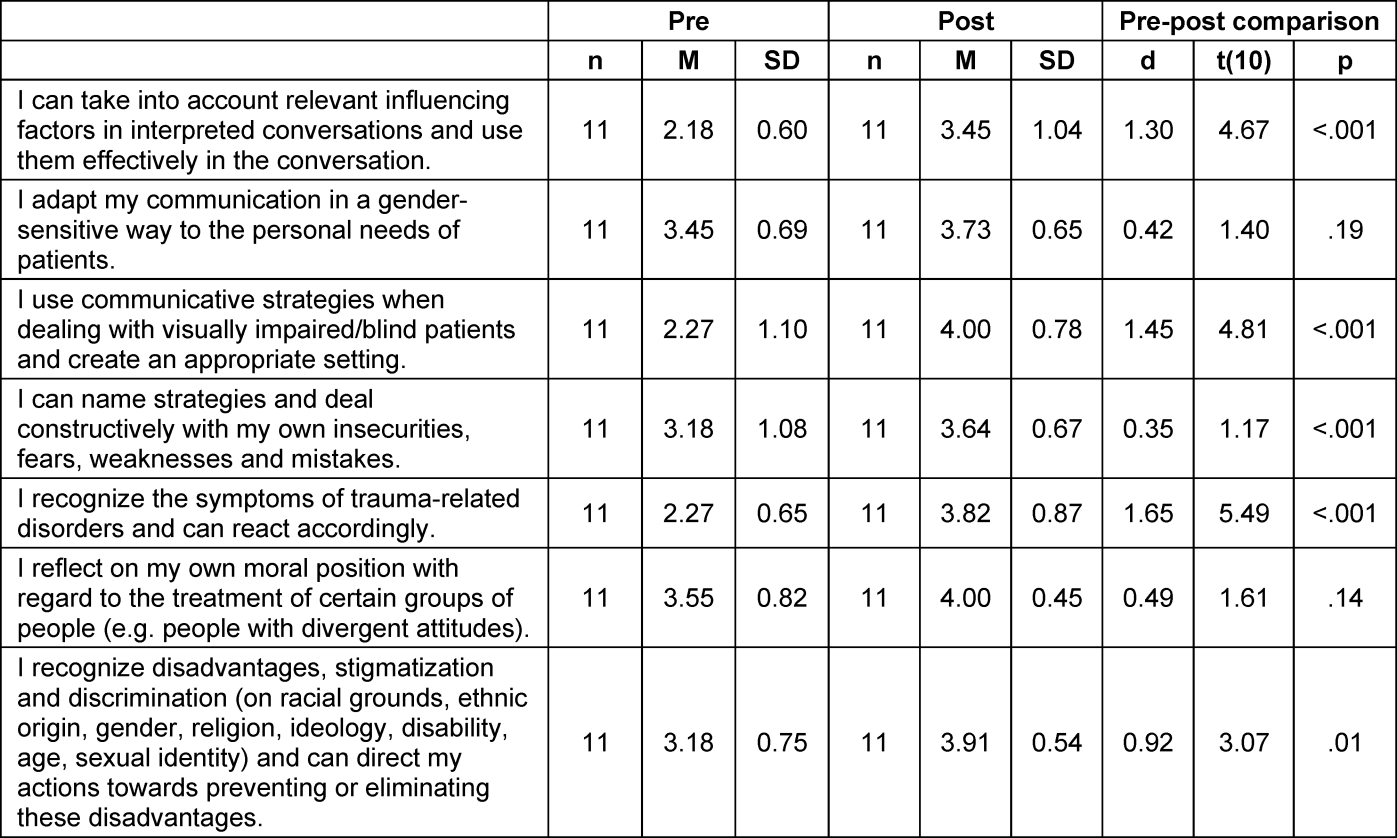
Subjective assessment of students’ competence before and after course participation, measured on a 5-point Likert scale (1=not competent; 5=very competent)
