# Supplementary material for: “I didn’t mean it that way…”: Design and evaluation of an elective course on dealing with discrimination in medical communication
Source: GMS J Med Educ. 2025 Jun 16;42(3):Doc40. doi: 10.3205/zma001764 (PMC12286879; doi:10.3205/zma001764)
Supplement: Learning objectives of the course based on the National Competence-Based Learning Objectives Catalog 2.0 [file JME-42-40-s-001.pdf]

Attachment 1: Learning objectives of the course based on National Competence-Based Learning Objectives Catalog 2.0

Overarching learning objectives:

- Self-knowledge, self-reflection, self-criticism and self-development (VIII.6-03.1.1, VIII.6-03.1.6, VIII.6-03.1.7)
- Dealing with discrimination (VIII.6 -04.4.13)
- Sociocultural diversity (VIII.2.05.1.2)
- Diversity & vulnerable groups (VIII.6 -04.1.2, VIII.6 -04.1.4)

Subject-specific learning objectives:

- Dealing with limited communication skills (VIII.2-05.4.1, VIII.2-05.4.2, VIII.2-05.4.3)
- Dealing with different insurance statuses (VIII.5-02.2.1)
- Gender-sensitive communication (VIII.2.05.1.2)
- Dealing with ethical conflicts (VIII.6-01.2.9)
- Dealing with language barriers and the use of language mediation (VIII.2.05.3.1, VIII.2.05.3.4)

Attachment 1 to Schwab L, Strauß B, Philipp S. *“I didn’t mean it that way…”: Design and evaluation of an elective course on dealing with discrimination in medical communication*. GMS J Med Educ. 2025;42(3):Doc40. DOI: 10.3205/zma001764
