# Supplementary material for: “I didn’t mean it that way…”: Design and evaluation of an elective course on dealing with discrimination in medical communication
Source: GMS J Med Educ. 2025 Jun 16;42(3):Doc40. doi: 10.3205/zma001764 (PMC12286879; doi:10.3205/zma001764)
Supplement: Example of a session [file JME-42-40-s-002.pdf]

Attachment 2: Example of a session

Topic: Dealing with right-wing extremist patients and believers in conspiracies

Time	Topic	Contents	Material
15'	Arrival	• Introduction round<br>• Presentation of the institution<br>• Expectation query<br>• Present schedule	Power-Point
30'	Introduction to the topic of right-wing extremism and misanthropy	• What is right-wing extremism?<br>• Figures and studies on the topic<br>• Narratives and forms of organization (especially in relation to medicine)<br>• Significance in the medical context	Power-Point<br>Menti
25'	Role and attitude	• What is my role, what is my attitude?<br>• Who is involved when patients make misanthropic or conspiracy-mongering statements?<br>• What are spaces for action? Where are boundaries?<br>• Support and assistance systems	Flipchart<br>Moderation cards
5'	Break		
30'	Collegial case consultation	• Discuss possible courses of action together in the group in a structured way using a real case	
45'	Conversation strategies with extreme right-wingers	• What are the functions of extreme right-wing and misanthropic attitudes<br>• What do these functions mean for conversation strategies?<br>• What are specific discussion strategies?	
10'	Break		

Attachment 2 to Schwab L, Strauß B, Philipp S. *“I didn’t mean it that way…”: Design and evaluation of an elective course on dealing with discrimination in medical communication*. GMS J Med Educ. 2025;42(3):Doc40. DOI: 10.3205/zma001764

30'	Exercise with simulation patients (SP)<br>“Belief in conspiracies, right-wing populist attitudes & vaccination”	The doctor should...<br>Perform a brief knee examination and discuss further treatment steps<br>Inquire about the patient's possible vaccination wishes and advise on vaccination<br>If necessary, use the CALM model (de-escalation) in conversation/apply conversation strategies when dealing with extreme right-wing people/conspiracy theorists<br>Other participants receive observation assignments	Role for SP<br>Information for doctors<br>Feedback form<br>Flipchart for observation tasks
35'	Feedback discussion of the exercise with SP	Reflection on exercise: which communicative strategies were used? How did the doctor respond to the patient’s attitudes?<br>What went well? What could be improved?<br>How did the doctor feel in the role? How did the observers feel?	
15'	Conclusion	• Self-reflection round<br>• How am I doing now?<br>• What went particularly well?<br>• What was a challenge for me today?<br>• What do I take away from today's meeting?<br>○ For me personally?<br>○ For my professional activity?<br>Feedback round on the unit	Worksheets

Attachment 2 to Schwab L, Strauß B, Philipp S. *“I didn’t mean it that way…”: Design and evaluation of an elective course on dealing with discrimination in medical communication*. GMS J Med Educ. 2025;42(3):Doc40. DOI: 10.3205/zma001764
