# Supplementary material for: “I didn’t mean it that way…”: Design and evaluation of an elective course on dealing with discrimination in medical communication
Source: GMS J Med Educ. 2025 Jun 16;42(3):Doc40. doi: 10.3205/zma001764 (PMC12286879; doi:10.3205/zma001764)
Supplement: Pre-evalatuation questionnaire [file JME-42-40-s-003.pdf]

Attachment 3: Pre-evaluation questionnaire

On the following pages, we will ask you questions about discrimination in a medical context and your experiences to date. The aim of this survey is to evaluate the course so that we can revise and improve it. To this end, we will ask you questions at the beginning and end of the event.

Participation in the study is voluntary and refusal to participate has no disadvantages. You can withdraw from the study at any time without giving reasons. All data collected as part of this study will be pseudonymized, evaluated anonymously and stored, i.e. your name will not be recorded and your data can no longer be assigned to your person. The data will be scientifically evaluated, whereby the evaluation will be summarized for all or some of the test subjects.

I am over 18 years old and have read the information. I agree to participate in the study.

Yes

No (study ends subsequently)

In order to evaluate and improve the event, we would like to compare how your answers at the beginning of the seminar have changed compared to the end of the seminar.

To do this, please create an individual test person code.

- The first two letters of your mother's first name (e.g. Kim = KI)
- Second letter of your own first name (e.g. Toni = O)
- The last two digits of your year of birth (e.g. 1994 = 94)
- The first two letters of your father's first name (e.g. Mohammed= MO)
- Complete code (e.g. KIO94MO)

Sociodemographics

1. Which gender do you feel you belong to?

Female

Male

Miscellaneous

Not specified

2. How old are you?

3. In which semester are you currently studying medicine?

4. Prior knowledge

First of all, we would like to know about your previous experience with the seminar topics. The questions relate to previous experience that you have gained during your medical studies, through professional experience and most recently in extracurricular experiences.

How much previous experience do you already have in the subject areas **from your medical studies**?

	No experience				A lot of experience
Communication-impaired persons (e.g. visually impaired)					
Persons without health insurance					
Trans* and/or non-binary people					
Refugees					
People with right-wing extremist attitudes and/or belief in conspiracy theories					
Use of language mediation in medical treatment (interpreters in presence/ via video/ telephone/ internet-based translation tools)					

How much **professional experience** do you already have with the following groups of people/settings (e.g. internships, work shadowing, ward days...)?

	No experience				A lot of experience
Communication-impaired persons (e.g. visually impaired)					
Persons without health insurance					
Trans* and/or non-binary people					
Refugees					
People with right-wing extremist attitudes and/or belief in conspiracy theories					
Use of language mediation in medical treatment (interpreters in presence/ via video/ telephone/ internet-based translation tools)					

How much **extracurricular experience** do you already have with the following groups of people/settings? (e.g. everyday life, leisure time, voluntary work...)

	No experience A lot of experience 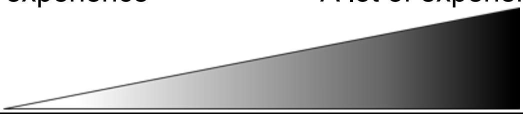				
Communication-impaired persons (e.g. visually impaired)					
Persons without health insurance					
Trans* and/or non-binary people					
Refugees					
People with right-wing extremist attitudes and/or belief in conspiracy theories					
Use of language mediation in medical treatment (interpreters in presence/ via video/ telephone/ internet-based means of translation)					

5. Attitude

To what extent do you agree or disagree with the following statements?

	1 = do not agree at all	*2 = do not agree*	*3 = partly*	*4 = agree*	*5 = fully agree*
E1: I find it very difficult to deal professionally with people who openly espouse right-wing extremist views. *(neg)*					
E2: For my future profession as a doctor, it is important to have dealt with the realities of different people's lives					
E3: To be able to work professionally as a doctor, I need to know and respect my personal needs and boundaries					
E4: I feel inhibited and unsure about interacting with visually impaired/blind patients. *(neg)*					
E5: Racist violence and experiences of discrimination can be understood as traumatization					
E6: If the use of professional language mediators is not possible during treatment, it is not a problem if relatives or friends of the patient take over the interpreting. *(neg)*					

E7: I find it difficult to communicate with patients whose gender I cannot clearly classify. *(neg)*					

6. Interest

How interested are you in the following topics?

	1 = not interested	*2 = hardly interested*	*3 = partly*	*4 = interested*	*5 = very interested*
I1: Addressing ethical dilemmas in medical care that arise due to individual health-related beliefs (e.g. belief in conspiracies) or certain political attitudes (e.g. far-right) of patients					
I2: Information about cultural misunderstandings or "culture" specific topics					
I3: Lifeworlds of trans* and non-binary people and how these shape interaction in the medical field					
I4: Dealing with the consequences of multiple experiences of discrimination in medicine and medical care					
I5: Connection between social inequality, health and illness (e.g. influence of ethnicity and experiences of racism)					
I6: Include self-awareness, self-reflection, self-criticism and self-development in my everyday professional activities					
I7: Learn more about the living environment of visually impaired/blind people					

7. Knowledge

How well informed do you feel about the following topics in general?

	1 = not informed	*2 = hardly informed*	*3 = partly*	*4 = somewhat informed*	*5 = very informed*
W1: Different job profiles and providers in language mediation, as well as the roles and tasks of all those involved in language-mediated communication					
W2: Strategies for communicating with people who believe in medical conspiracy theories					
W3: Barriers experienced, possible concerns and wishes of trans* people and tasks of doctors and the healthcare system in relation to trans* people					
W4: The elements of a patient-centered attitude (congruence, acceptance, empathy)					
W5: Communicative strategies in dealing with visually impaired/blind patients					
W6: Central symptoms after traumatic experiences (e.g. post-traumatic stress disorder) and their effects and special needs of those affected in medical treatment					
W7: Procedure for patients and persons with illegal residence status who do not have health insurance					

Attachment 3 to Schwab L, Strauß B, Philipp S. *“I didn’t mean it that way…”: Design and evaluation of an elective course on dealing with discrimination in medical communication*. GMS J Med Educ. 2025;42(3):Doc40. DOI: 10.3205/zma001764

8. Competence

How competent do you consider yourself in the following areas?

	1 = not competent	*2 = hardly competent*	*3 = partly*	*4 = competent*	*5 = very competent*
K1: I can take into account relevant influencing factors in interpreted conversations and use them effectively in the conversation.					
K2: I adapt my communication to the personal needs of patients in a gender-sensitive way.					
K3: I use communicative strategies when dealing with visually impaired/blind patients and create an appropriate setting.					
K4: I can name strategies and deal constructively with my own insecurities, fears, weaknesses and mistakes.					
K5: I recognize the symptoms of a trauma-related disorder and can react accordingly.					
K6: I reflect on my own moral position with regard to the treatment of certain groups of people (e.g. people with divergent attitudes).					
K7: I recognize disadvantages, stigmatization and discrimination (on racial grounds, ethnic origin, gender, religion, ideology, disability, age, sexual identity) and can direct my actions towards preventing or eliminating these disadvantages					

9. Your expectations of the seminar

What are you interested in with regard to the individual thematic focuses? Which questions would you like to have answered by the end of the event?

- Trauma and discrimination-sensitive treatment
- Trans*people in medical communication
- People with right-wing extremist attitudes and conspiracy beliefs
- Patients without health insurance
- Use of language mediation
- more

Attachment 3 to Schwab L, Strauß B, Philipp S. *“I didn’t mean it that way…”: Design and evaluation of an elective course on dealing with discrimination in medical communication*. GMS J Med Educ. 2025;42(3):Doc40. DOI: 10.3205/zma001764
