# Supplementary material for: “I didn’t mean it that way…”: Design and evaluation of an elective course on dealing with discrimination in medical communication
Source: GMS J Med Educ. 2025 Jun 16;42(3):Doc40. doi: 10.3205/zma001764 (PMC12286879; doi:10.3205/zma001764)
Supplement: Post-evaluation questionnaire [file JME-42-40-s-004.pdf]

Attachment 4: Post-evaluation questionnaire

The event is currently still a pilot project that we would like to revise and improve in order to be able to establish it in the medical curriculum in the long term.

Your participation in the evaluation is therefore very important!

We would like to ask you a few questions below about your expectations, prior knowledge and level of knowledge to date. The survey will take about 10 minutes.

I agree to participate in the study. My participation is voluntary. I am aware that I can withdraw my consent at any time without giving reasons with effect for the future. Furthermore, I agree that my personal data may be published in an anonymized form and shared with and disclosed to other researchers.

I am over 18 years old and have read the information. I agree to participate in the study.

No (study ends afterwards)

In order to evaluate and improve the event, we would like to compare how your answers at the beginning of the seminar have changed compared to the end of the seminar.

To do this, please create an individual test person code.

- The first two letters of your mother's first name (e.g. Kim = KI)
- Second letter of your own first name (e.g. Toni = O)
- The last two digits of your year of birth (e.g. 1994 = 94)
- The first two letters of your father's first name (e.g. Mohammed= MO)
- Complete code (e.g. KIO94MO)

1. Have you already taken part in the inline survey on the first date?

Yes (skips sociodemographics)

No (continue with sociodemographics)

Sociodemographics

2. Which gender do you feel you belong to?

Female

Male

Miscellaneous

Not specified

3. How old are you?

In which semester are you currently studying medicine?

4. Attitude

To what extent do you agree or disagree with the following statements?

	1 = do not agree at all	*2 = do not agree*	*3 = partly*	*4 = agree*	*5 = fully agree*
E1: I find it very difficult to deal professionally with people who openly espouse right-wing extremist views. *(neg)*					
E2: For my future profession as a doctor, it is important to have dealt with the realities of different people's lives					
E3: To be able to work professionally as a doctor, I need to know and respect my personal needs and boundaries					
E4: I feel inhibited and unsure about interacting with visually impaired/blind patients. *(neg)*					
E5: Racist violence and experiences of discrimination can be understood as traumatization					
E6: If the use of professional language mediators is not possible during treatment, it is not a problem if relatives or friends of the patient take over the interpreting. *(neg)*					
E7: I find it difficult to communicate with patients whose gender I cannot clearly classify. *(neg)*					

5. Interest

How interested are you in the following topics?

	1 = not interested	*2 = hardly interested*	*3 = partly*	*4 = interested*	*5 = very interested*
I1: Addressing ethical dilemmas in medical care that arise due to individual health-related beliefs (e.g. belief in conspiracies) or certain political attitudes (e.g. far-right) of patients					
I2: Information about cultural misunderstandings or "culture" specific topics					
I3: Lifeworlds of trans* and non-binary people and how these shape interaction in the medical field					
I4: Dealing with the consequences of multiple experiences of discrimination in medicine and medical care					
I5: Relationship between social inequality, health and illness (e.g. influence of ethnicity and experiences of racism)					
I6: Include self-awareness, self-reflection, self-criticism and self-development in my everyday professional activities					
I7: Learn more about the living environment of visually impaired/blind people					

Attachment 4 to Schwab L, Strauß B, Philipp S. *"I didn't mean it that way…": Design and evaluation of an elective course on dealing with discrimination in medical communication*. GMS J Med Educ. 2025;42(3):Doc40. DOI: 10.3205/zma001764

6. Knowledge

How well informed do you feel about the following topics in general?

	1 = not informed	*2 = hardly informed*	*3 = partly*	*4 = somewhat informed*	*5 = very informed*
W1: Different job profiles and providers in language mediation, as well as the roles and tasks of all those involved in language-mediated communication					
W2: Strategies for communicating with people who believe in medical conspiracy theories					
W3: Barriers experienced, possible concerns and wishes of trans* people and tasks of doctors and the healthcare system in relation to trans* people					
W4: The elements of a patient-centered attitude (congruence, acceptance, empathy)					
W5: Communicative strategies in dealing with visually impaired/blind patients					
W6: Central symptoms after traumatic experiences (e.g. post-traumatic stress disorder) and their effects and special needs of those affected in medical treatment					
W7: Procedure for patients and persons with illegal residence status who do not have health insurance					

7. Competence

How competent do you consider yourself in the following areas?

	1 = not competent	*2 = hardly competent*	*3 = partly*	*4 = competent*	*5 = very competent*
K1: I can take into account relevant influencing factors in interpreted conversations and use them effectively in the conversation.					
K2: I adapt my communication to the personal needs of patients in a gender-sensitive way.					
K3: I use communicative strategies when dealing with visually impaired/blind patients and create an appropriate setting.					
K4: I can name strategies and deal constructively with my own insecurities, fears, weaknesses and mistakes.					
K5: I recognize the symptoms of a trauma-related disorder and can react accordingly.					
K6: I reflect on my own moral position with regard to the treatment of certain groups of people (e.g. people with divergent attitudes).					
K7: I recognize disadvantages, stigmatization and discrimination (on racial grounds, ethnic origin, gender, religion, ideology, disability, age, sexual identity) and can direct my actions towards preventing or eliminating these disadvantages					

8. Presence

How many sessions of the event series did you attend?

1
2
3
4
5
6

9. Motivation

My motivation at the beginning of the seminar was…

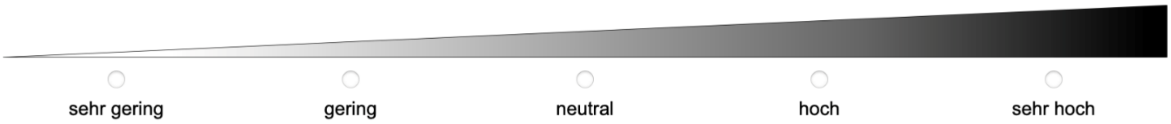

(en: very low, low, neutral, high, very high)

My motivation at the end of the seminar was…

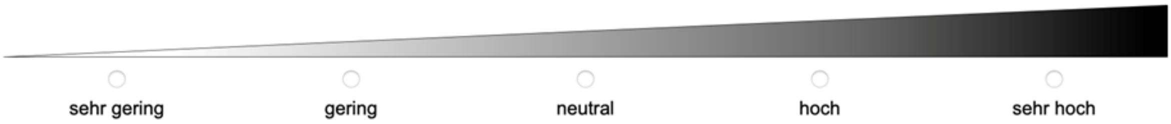

(en: very low, low, neutral, high, very high)

10. Relevance

How do you rate the relevance of the individual learning content for practice?

	1= not relevant	2 = little relevant	3 = relevant	4= very relevant
Role/attitude of a doctor				
Communication-impaired patients				
Patients without health insurance, special circumstances				
Trauma-sensitive communication using the example of racism				
Needs of trans* in medical communication				
Dealing with right-wing extremists and conspiracy theorists				
Language mediated treatment				

11. All in all, attending the event was worthwhile for me

stimme überhaupt nicht zu	stimme nicht zu	stimme teils/teils zu	stimme zu	stimme voll und ganz zu
○	○	○	○	○

(en: strongly disagree, disagree, party agree, agree, strongly agree)

12. What grade would you give the elective subject overall?
    1
    2
    3
    4
    5
    6

13. What topics did you miss in the event?
14. Further comments/thoughts
15. What did you remember most about the seminar series?

Attachment 4 to Schwab L, Strauß B, Philipp S. *“I didn’t mean it that way…”: Design and evaluation of an elective course on dealing with discrimination in medical communication*. GMS J Med Educ. 2025;42(3):Doc40. DOI: 10.3205/zma001764
